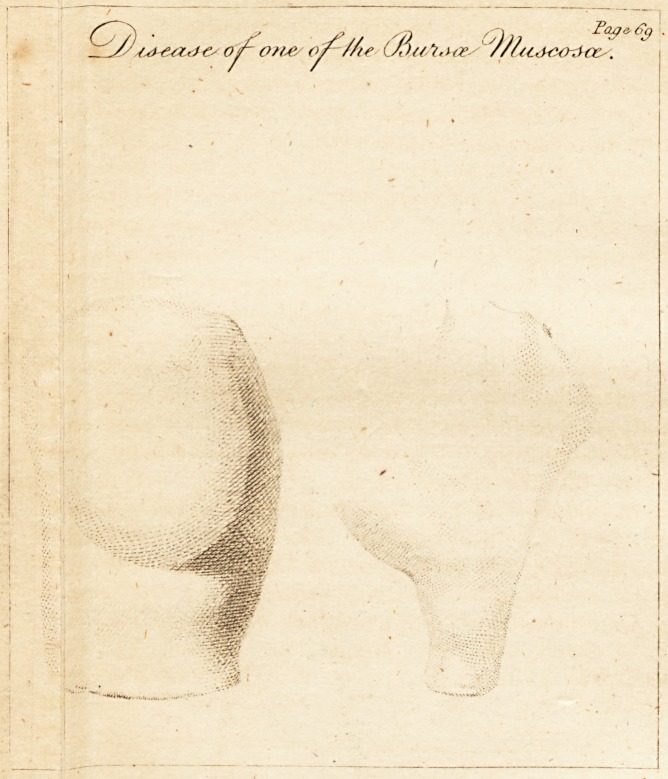# Disease of One of the Bursæ Mucosæ

**Published:** 1803-07-01

**Authors:** 


### Disease *of one of the* Bursæ Mucosæ.

[ With an Engraving. ]

IN the course of last winter, while engaged in dissecting the bursæ mucosæ of the human body, I discovered two new bursæ on the knee, and one on the elbow joint.

A description of these sacs will, I hope, be not uninteresting, particularly as I have since found the former of them to be frequently the seat of a disease, which in its early stage might be mistaken for one of the symptoms of white swelling of this joint, and which in its advanced stage has, I suspect, been generally considered as an encysted tumour.

An incision made through the integuments from the upper edge of the patella to the knob of the tibia, discloses two bursæ mucosæ; the superior of these sacs is seated on the upper part of this patella, the inferior does not reach so low down as the lower joint of the bone. These sacs are separated from each other by a common transverse partition, in which I have not found any hole of communication. The parietes and glairy contents of these sacs very strongly resemble those of the other bursæ mucosæ; so that we cannot doubt but that they are of the same nature with the other bursæ, although they differ in their situation as not being interposed between tendons, or between tendon and bone.

These bursæ on the patella are equally, and in my mind, more liable than other bursæ to an accumulation of their natural fluid, causing a disease productive of much distress to the patient. A knowledge of this disease will assist us in discriminating the various diseases of this joint, and may rescue the patient, in many instances, from that severity of treatment which is generally adopted in incipient white swellings. As this accumulation presents very different appearances in its incipient and in its advanced stages, I shall speak of each stage separately.

*In the incipient stage*, a tumour, the size of a wallnut, is to be seen on the fore part of the knee, below the middle height of the patella. By a slight attention it is ascertained that this tumour is seated above, and not under the ligamentum patellæ. This tumour gives to the finger neither a sense of fluctuation nor of elasticity, it readily yields to the finger, and indeed more readily than I recollect any other tumor to yield; the sensation to the finger is as if the tumour changed its place. On removing the finger no pitting remains, the tumour instantly regaining its former appearance. The tumour in this stage is subject to an occasional increase and decrease; by what local or constitutional circumstances these changes are induced, I have not been able to ascertain. The patient is free from pain except when he attempts to bend the knee much, or to kneel, and this posture is very distressing to him.

I cannot undertake to say how long the disease may remain in this state; I have seen some cases where it had continued for eighteen or twenty months.

This stage of the disease much resembles a symptom frequently attending white swelling of the knee; the symptom I allude to, is a swelling which appears between the lower point of the patella and knob of the tibia, and which is seated in the bursa that lies *under* that ligament. By attending to the situation of these two tumours we can discriminate with certainty. For the cure of this accumulation in the superficial bursa, (at this stage) I have tried frictions with various stimulating liniments, but cannot say that my patients derived material or permanent benefit from the practice. I have also advised pressure by means of sheet-lead and a tight bandage; this practice, in one case, caused a rupture of the bursa, an effusion of its contents into the surrounding cellular and ligamentous substance, and I believe effected a perfect cure, at least the patient did not return.

All the patients whom I have seen labouring under this stage of the disease, have been among the working class, and a very large majority have been servants of the female sex. Although this disease does not endanger life, nor (when its nature is known) threaten the loss of the joint, or even induce constant pain, yet as it prevents these poor people from earning a subsistence in, perhaps, the only mode of which they are capable, it must on this account alone attract the attention of the humane practitioner. We trust therefore that the exertions of humanity and science will soon prove successful in discovering an effectual cure for this wretched class of patients.

I have met with not a few instances of what I term the second stage of this disease, or the state of extreme accumulation of synovial fluid in these bursæ, and from them I deduce the following characters of this disease.

A globular tumour larger than a hen's egg, is seen on the fore part of the knee; integuments covering the tumour at first are not discoloured, but in the latter periods of the disease these become red and inflamed. The patient suffers more more or less uneasiness in kneeling, and some are altogether incapable of this posture. The tumour undergoes occasional increases and decreases of size. The causes of these changes I am yet unacquainted with. An obscure fluctuation can be felt. The patella is almost concealed by the tumour; upon a close examination, however, the upper edge of this bone will be felt, and will be found to enjoy its usual mobility. When the patella is moved by the fingers, the tumour does not seem to be moved equally with it. The inflammation of the integuments is attended with a good deal of pain, and generally terminates by an ulceration; this communicating with the cavity of the sac, allows a large quantity of glairy fluid to escape; and a large dense slough is in a few days cast off; after which the glairy discharge daily decreases, and the part completely heals; more frequently, however, a small discharge continues to ooze through the orifice, now become callous. In one instance I saw the inflammation of the integuments dispersed by the application of aq. lithargyri acetati, although it had been so far advanced as to make me suspect an ulceration and opening of the sac.

When the ulceration of the integuments and bursting of the sac is about to take place, the integuments will be found most thin at the very apex of the tumour.

This stage of the disease has been, as I suspect, generally considered and treated as an encysted tumour. If the foregoing description accurately mark the disease, we shall at least enjoy the satisfaction of treating a disease, whose seat we know to be limited, (to these bursæ) and whose inflammation probably will not spread to the cavity of the joint; an apprehension which, I doubt not, has induced the practitioner frequently to leave this disease to its natural cure, the spontaneous bursting of the sac. The patient will thus be more speedily relieved from his disease, and the practitioner will administer that relief with perfect security.

In this advanced stage of the disease we cannot hope to effect a removal of the tumour by any topical or constitutional remedies; although we may sometimes succeed in resolving the inflammation of the skin, and thus for the time prevent the bursting of the sac.

The only mode of treatment which I have seen successfully used was, making a small incision into the tumor. In the last case where this treatment had been used, the incision was made, not into the apex, but in the inferior (or most depending) part; and I remarked, that after the third day from the incision, the glairy fluid could not be pressed out by applying the fingers in the vicinity of the incision, while it flowed in large quantity if the pressure were applied to the apex of the tumour.

From this circumstance I have been induced to think that the tumour may with more advantage be opened by an incision made into its apex; for thus we shall obtain an uninterrupted drain for the fluid, and we also hope that the lining membrane of the cavity will more readily and more speedily be affected by inflammation, and thus the cavity be completely obliterated.

Query, Would it not be advisable to attempt the cure of this stage by the same means as are used for the cure of Hydrocele, by injection? We are not to apprehend a higher degree of inflammation from this treatment, than would attend an incision; and even though a higher degree of inflammation should follow, we shall not be under apprehension that the cavity of the joint will be engaged in it. Besides, we have reason to hope, that the inflammation excited by the injection will be sufficient, with the compression of a roller, to cause an accretion of the sides, and an obliteration of the cavity of the sac.

Having discovered these bursæ on the patella, I was led by analogy to suspect the existence of similar sacs on that part of the ulna where it is covered only by the common integuments. By making a longitudinal incision through the integuments of this part, I readily discovered a bursa, the capacity of which is such as would contain a body larger than a filbert nut.

I have seen only one instance of disease in this bursa. The patient laboured under venereal pains of his limbs; and in the site of this bursa, on one arm, was a tumour the size of a pigeon's egg, and which felt as if filled with a soft fatty substance. This tumour yielded with his other venereal syptoms to the use of mercury.

From the situation of these bursæ, on bones covered only by the common integuments, we may infer, that the use of these sacs is more extensive than has been hitherto supposed, as they seem to protect the skin from the effect of pressure against the bone.

*Dublin, May 12th.* 1803. X.

P.S. The drawing exhibits two views of the alteration induced in the form of the joint by the second stage of this disease. The drawing was taken from a cast in plaister of Paris.

*To*

*Arneman's Instruments for extracting the Cataract.*

Page 65

Page 69

*Disease of one of the Bursæ Muscosæ.*

## Figures and Tables

**Figure f1:**